# Beyond labels: determining the true type of blood gas samples in ICU patients through supervised machine learning

**DOI:** 10.1186/s12911-025-03115-3

**Published:** 2025-07-24

**Authors:** Johan Helleberg, Anna Sundelin, Johan Mårtensson, Olav Rooyackers, Ragnar Thobaben

**Affiliations:** 1 Perioperative Medicine and Intensive Care, Karolinska University Hospital, Stockholm, Sweden; 2 Section of Anaesthesiology and Intensive Care Medicine, Department of Clinical Sciences, Intervention and Technology (CLINTEC), Karolinska Institutet, Stockholm, Sweden; 3 Section of Anaesthesiology and Intensive Care Medicine, Department of Physiology and Pharmacology, Karolinska Institutet, Stockholm, Sweden; 4 Division of Information Science and Engineering, Digital Futures Faculty, School of Electrical Engineering and Computer Science, Royal Institute of Technology (KTH), Stockholm, Sweden

**Keywords:** Supervised machine learning, Biochemistry, Blood gas, Intensive care, Pediatric, Adult, ICU

## Abstract

**Background:**

In the Intensive Care Unit (ICU), data stored in patient data management systems (PDMS) is commonly used in clinical practice and research. Parameters from point-of-care arterial blood gas (BG) analysis are used in the diagnosis and definition of syndromes such as sepsis and ARDS, but manual entry of the blood source (arterial or venous) into the PDMS introduces the risk of mislabeling venous samples as arterial. Our study aimed to employ supervised machine learning to accurately identify blood gas samples as arterial or venous using PDMS data.

**Methods:**

A retrospective, single-center observational cohort study including all blood gases during 2018 from a Swedish, pediatric and adult general ICU. Chemical parameters from BG analysis and clinical parameters such as mean arterial pressure (MAP) and saturation (SpO2) were utilized as features. A specialist physician in Intensive Care manually determined the true class of each sample through comprehensive retrospective chart review. The samples were split into training, testing and holdout sets. Training was performed using cross-validation in the training set, with forward stepwise feature selection and Bayesian hyperparameter optimization, and accuracy was assessed using area under the precision recall curve (AUCPR) in the test set. The best model was compared to a multivariate logistic regression model (LR) in the holdout set.

**Results:**

Among 33,800 samples (30,753 arterial, 3,047 non-arterial) from 691 ICU admissions, 150 (0.44%) were erroneously marked. The best performing algorithm was extreme gradient boosting (XGboost) using 9 features, with an AUCPR of 0.9974 (95% CI 0.9961–0.9984), significantly better than the LR model (AUCPR = 0.9791, 95% CI 0.9651–0.9904).

**Conclusion:**

Supervised machine learning demonstrates efficacy in determining blood gas sample type from ICU patients. This approach shows promise for improving the accuracy of research and clinical applications relying on blood gas data.

**Supplementary Information:**

The online version contains supplementary material available at 10.1186/s12911-025-03115-3.

## Background

Data from the Intensive Care Unit (ICU) stored in patient data management systems (PDMS) is increasingly used in clinical practice and research. Large multinational collaborations use data driven methodology to define and describe patient groups and diagnoses, most notably the Sepsis 3-criteria [1] for adult patients and the Phoenix [2] score for pediatric sepsis. Automated systems based on these definitions are being integrated into ICUs for surveillance [3] and clinical decision support [4, 5].

Biochemical parameters from point-of-care blood gas analysis are commonly used as features in these definitions. In clinical practice blood for analysis could come from many different sources, e.g. arterial, venous, central venous, or capillary. Sample type is typically recorded at the point of care. Manual errors leading to mislabeling of venous samples as arterial or vice-verse has been described [4, 6]. Moreover, human errors in the ICU are not entirely random, but correlate to the number of patient activities performed by bedside clinicians [7, 8] and illness severity [9]. The distribution of labeling errors could be related to these factors.

Using data with this type of error could lead to flawed conclusions, particularly when using parameters that are known to vary between arterial and venous blood, e.g. hemoglobin oxygen saturation (SO_2_), pH, arterial partial pressure of oxygen (PaO_2_), or any parameter derived from those, e.g. the ratio of arterial oxygen tension to inspired oxygen fraction (PaO_2_/ F_i_O_2_ or PF-ratio). These parameters are used extensively for diagnostic and prognostic purposes in the ICU, including quantifying organ failure according to Sequential Organ Failure Assessment Score (SOFA) [10] and grading Acute Respiratory Distress Syndrome (ARDS) [11]. Additionally, overestimation of SO_2_ based on peripheral oxygen saturation measured by plethysmography (SpO_2_) is associated with worse outcomes and delayed care delivery [12], and data driven studies are warranted on predicting this phenomenon, described as silent hypoxemia [13, 14]. Therefore, the development of an accurate automated system for classification of blood gases in the ICU PDMS setting has many potential benefits.

We aimed to develop and validate a supervised machine learning (ML) model to differentiate between arterial and non-arterial blood gas samples from ICU patients in PDMS data with the best possible accuracy and sought to determine which class of ML model is most suitable for this task and how to optimize such a model to be used in large ICU datasets to detect erroneously labeled blood gas samples.

## Methods

### Data extraction

We conducted a retrospective, single-center cohort study. All point-of-care blood gas samples labeled as arterial, venous, or central venous from adult and pediatric patients treated in the ICU at Karolinska University Hospital, Huddinge, between January 1 and December 31, 2018, were extracted and included from the Patient Data Management System (PDMS; Centricity Critical Care, GE Healthcare, Chicago, IL, USA). Samples were analyzed on two ABL800 Flex blood gas analyzers (Radiometer Medical A/S, Brønshøj, Denmark), with sample type manually entered by the clinician at the time of analysis. Samples with technical flaws that prevented analysis (e.g. hemolysis, inadequate sample volume) were rejected by the blood gas analyzer and were not transmitted to the PDMS; no other exclusions were made. Clinical data were extracted from the PDMS and EHR (TakeCare, CompuGroup Medical CGM, Koblenz, Germany).

### Data partitioning and features

To enable unbiased final model evaluation, a random subset of patient admissions representing at least 20% of all blood gas samples was selected as a holdout set prior to manual labeling and data analysis. The approximately 80% remaining samples were used as development set for feature engineering and model training. The development set was further split randomly 80%/20% into a training and testing set, to evaluate model performance during development prior to a final validation in the holdout set. Using 80% for model development while using 20% for final holdout was chosen to allow a large dataset in training to maximize model performance while still allowing robust evaluation, a common strategy in machine learning [15].

The variables included were pH, partial pressure of carbon dioxide in blood (pCO_2_), partial pressure of oxygen (pO_2_), Base Excess (BE), Standard Bicarbonate (StHCO_3_), Anion Gap, SO_2_, partial pressure corresponding to 50% hemoglobin oxygen saturation (p50), fraction of Methemoglobin (FMetHb), hemoglobin concentration (Hb), Hematocrit (Hct), and concentrations of Sodium (Na), Potassium (K), Calcium (Ca), Chloride (Cl), Glucose, and Lactate. pH, sO2, pO2, BE, pCO2 were calculated using Severinghaus’ [16] formula, or Siggaard-Andersen’s [17] equations, or numeric inversions thereof [18], if any one of them were missing and the required variables for calculation were present (Supplementary Sect. 2).

Clinical variables included the median SpO2 during the ten minutes before sampling and the closest matching SpO_2_ by value within +/- 10 min from blood gas sampling, and the differences between SO_2_ and these SpO_2_ values. The highest and lowest mean arterial blood pressure (MAP) and SpO_2_ within +/- 10 min from sampling were recorded. F_i_O_2_-setting at 5 min prior to and 15 min after blood gas sampling were recorded and the P/F-ratio was calculated from SO2 and Fio2 at 5 min prior.

Missing values in the MAP values (likely indicating absence of arterial line), changes in F_i_O_2_ from before to after blood gas sampling (likely indicating a bedside action based on arterial saturation or PO_2_), and an SO_2_ between the lowest and highest measured SpO_2_ within +/- 10 min (indicating that the SO_2_ was similar to the currently measured SpO_2_-values) were considered possible candidate predictors for blood gas type and encoded as categorial variables. See supplementary Table 1 for full variable definitions and rationale.

### True classes of samples

The true sample type was determined through manual review and interpretation of all blood gas samples by a specialist physician in Anesthesia and Intensive Care. Data was presented in a spreadsheet (Excel, Microsoft, Seatle, WA, USA) and the reviewer had access to all filtering, analysis and sorting tools in that software to aid classification. Clinical data from the PDMS and EHRS systems, including diagnosis, therapeutic procedures, administered medications and other information such as the original sample type, was also available.

If a sample was suspected to be mislabeled, a second intensivist independently reviewed the case, and a final classification was determined through consensus between the two reviewers. Additionally, all samples labeled as arterial with a recorded pO2 < 6.66 kPa (50 mmHg) were reviewed by both intensivists, regardless of whether mislabeling was suspected by the first reviewer. The final classification (arterial, or non-arterial) was considered the ground truth for model development.

### Data Preparation and feature engineering

In the training cohort, the predictive performance of each individual feature was assessed using a 5-fold cross-validated (CV) area under the receiving operator characteristic curve (AUROC) with the true class as the outcome. Features with a CV AUROC exceeding 0.75 had a 24-hour tri-cubic time-weighted mean calculated. The time-weighted mean difference (TMWD), representing the disparity between the feature value and its time-weighted mean, was then computed and considered as a potential feature.

### Imputation and dimensionality reduction

Missing values were imputed individually per dataset using the mean (for continuous features) or mode (for categorical features). The features were mean centered and scaled to unit variance.

In the training set, Pearson’s correlation coefficient was calculated between all features. If features had a higher correlation than 0.75 with any other feature, a principal component (PC) analysis of such correlated features was performed. Best subset selection in multivariate logistic regression with sample type as dependent parameter and using the Bayesian information criterion was used to determine which PCs to include. Those PCs were then calculated in each dataset and used as features instead of the underlying correlated variables.

Unsupervised dimensionality reduction was performed with t-distributed Stochastic Neighbor Encoding (t-SNE) [19] to visually determine if a low-dimensional representation of the data could produce separation of the classes before and after dimensionality reduction.

### Model training and selection

Due to expected class imbalance (approximately a 10:1 ratio of arterial to non-arterial samples) the area under the precision-recall curve (AUCPR) was used as the primary performance metric throughout the training procedure.

In the training set, a range of machine learning algorithms was trained and tuned using grid search over each algorithm’s hyperparameter space, with five-fold cross-validation (CV) applied to reduce the risk of overfitting [17]. The algorithms evaluated were random forest (RF), eXtreme Gradient Boosting (XGBoost), support vector machine (SVM), a feed-forward neural network (NN), regularized linear discriminant analysis (RDA), k-nearest neighbors (kNN), and logistic regression (LR) [18–24]. The two best performing algorithms, defined by the highest AUCPR in the testing set, were selected for further refinement.

For the two best performing algorithms after this grid search, a forward stepwise feature selection process was performed. At each number of features, a Bayesian Optimization process was used to find optimal hyperparameters for each algorithm. The performance on the test set was sequentially evaluated during this process, and if no improvement in AUCPR was seen after three rounds, the process was terminated and the model with the best performance was selected.

Finally, all models from the grid search (using the full feature set) and the two best models from the forward feature search were trained on the full training dataset and tested on the holdout set. See supplementary Sect. 3.

### Statistical analysis

Model performance was evaluated with AUCPR for overall predictive power, AUROC for discrimination, and the Brier score for calibration. Confidence intervals were estimated using bootstrapping for precision-recall (PR) curves and DeLong’s method for ROC curves [25]. Given the clinically grounded expectation that model output probabilities would cluster near 0 or 1, calibration was assessed visually using a Locally Estimated Scatterplot Smoother (LOESS) plot to avoid binning artifacts and allow a smooth, non-parametric estimation predicted vs. observed probabilities. Logistic regression was used to assess calibration-in-large.

The optimal classification threshold was determined using the Fβ score with β = 0.5, prioritizing high precision over recall to reduce false positives. Normality of variables was assessed using Q-Q plots. Welch’s t-test and the Wilcoxon rank-sum test were applied for comparisons between continuous variables, as appropriate. Chi-square test was used for comparisons of categorical variables. A p-value < 0.05 was considered statistically significant, with Bonferroni correction applied to account for multiple comparisons when applicable.

T-distributed stochastic neighbor embedding (t-SNE) was used to visualize class separation in the feature space before and after dimensionality reduction. Feature importance in the final model was evaluated using Shapley Additive Explanations (SHAP) [26, 27].

R version 4.2.1 with the packages caret, data.table, ggplot2, rtsne, Rcpp and C + + 14 with the Boost library was used for all calculations.

### Ethical approval

Swedish Ethical Review Authority (approval number 2019–06203, amendments 2022-04189-02 and 2024-01320-02) with a waiver of informed consent.

## Results

A total of 33,800 blood gas samples (30753 arterial, 3047 non-arterial) from 691 intensive care admissions were included. The development set consisted of 542 admissions with a total of 26,986 samples (24463 arterial, 2523 non-arterial) and the holdout set consisted of 149 admissions, with a total of 6814 samples (6186 arterial, 628 non-arterial). The number of samples per admission ranged between 1 and 818. Out of 691 admissions, 80 (11%) had at least one mislabeled blood gas in the PDMS system during their ICU stay. The total number of erroneously marked blood gas samples was 150 (0.44%). Patients with at least 1 error during their ICU stay had a significantly higher mean Simplified Acute Physiology Score (SAPS3) at admission compared to those who had no errors made (65.14 vs. 60.46, *p* = 0.019). The cohorts were evenly matched in most aspects, except that the holdout set contained fewer male patients and more patients with surgery prior to admission, (clinical characteristics in Table 1).

**Table 1:** Patient characteristics per set

Variable	Development (*n* = 542)	Holdout (*n* = 149)	*p*
Age	61.5 (0–93)	61 (0–87)	*p* = 0.362
Male Sex	322 (59.41%)	73 (48.99%)	*p* = 0.029
SAPS3 points	61.49 (+/- 18.06)	59.01 (+/- 17.42)	*p* = 0.152
Surgery prior to admission	145 (26.75%)	56 (37.58%)	*p* = 0.013
ARDS at admission	56 (10.33%)	13 (8.72%)	*p* = 0.671
Shock at admission	123 (22.69%)	36 (24.16%)	*p* = 0.789
IMV during ICU stay	277 (51.11%)	88 (59.06%)	*p* = 0.103
CRRT during ICU stay	79 (14.58%)	23 (15.44%)	*p* = 0.895
Death in ICU	69 (12.73%)	20 (13.42%)	*p* = 0.932

Data presented as median (range) or count (%). SAPS3 = Simplified acute physiology score, ARDS = acute respiratory distress syndrome, imv = invasive mechanical ventilation, crrt = continuous renal replacement therapy

Of all 39 candidate predictors defined, all but 4 differed significantly between the two classes of blood gas sample in univariate testing (supplementary Table 2) after adjusting for multiple testing. The 19 features with the best 5-fold CV AUROC for blood gas type in the development cohort are shown in Table 2. All of them had an AUROC of at least 0.5673 in univariate testing. No variable was missing in more than 6.63% of cases. The full descriptive statistics including ranges of all blood gas parameters are found in the supplement (Supplementary Table 3), split by adult or pediatric patients and arterial or venous.

**Table 2:** The ranges per sample class of the subset of candidate features with CV AUC greater than the median among the 39 candidate predictors in univariate logistic regression in the development cohort

Feature		Venous or central venous (*n* = 3151)	Arterial (*n* = 30649)	*p*	Missing
pH		7.38 (7.32–7.42) [6.76–7.61]	7.42 (7.37–7.46) [6.68–7.81]	*p* < 0.001	243 (0.72%)
pCO2 (kPa)		5.69 (5.09–6.63) [2.13–21.1]	5.05 (4.46–5.84) [1.08–24.7]	*p* < 0.001	260 (0.77%)
pO2 (kPa)		4.98 (4.35–5.72) [2–17.2]	10.9 (9.64–12.5) [1.05–75.8]	*p* < 0.001	0 (0%)
SO2 (%)		68.8 (61.8–75.1) [12–99.2]	96.1 (94.3–97.4) [3.5–100.1]	*p* < 0.001	0 (0%)
p50 (kPa)		3.85 (3.7–4) [0–9.19]	3.45 (3.28–3.67) [2.09–12.12]	*p* < 0.001	1542 (4.56%)
Cl (mmol/L)		106.49 (+/- 5.69) [70–136]	108.15 (+/- 5.4) [70–140]	*p* < 0.001	290 (0.86%)
SpO2 (max) (%)		98 (96.16–100) [38–100]	98 (96–99) [28–100]	*p* < 0.001	1161 (3.43%)
SpO2 - SO2-diff (med) (%)		27.7 (21.4–34.4) [-6.1–87]	0.5 (-0.7–1.7) [-50–88.5]	*p* < 0.001	1443 (4.27%)
SpO2 - SO2-diff (match) (%)		27 (20.5–33.8) [-18.7–88]	0.4 (-0.5–1.4) [-44.9–66]	*p* < 0.001	1161 (3.43%)
SO2 between SpO2 min/max	No	2988 (94.83%)	16,393 (53.49%)		
SO2 between SpO2 min/max	Yes	163 (5.17%)	14,256 (46.51%)	*p* < 0.001	0 (0%)
Age (Years)		58 (36–66) [0–93]	63 (49–69) [0–93]	*p* < 0.001	0 (0%)
PF-ratio (kPa)		16.3 (11.8–21.02) [3.65–81.9]	34 (23.21–44.8) [3.74–291.7]	*p* < 0.001	913 (2.7%)
MAP missing	No	1677 (53.22%)	29,904 (97.57%)		
MAP missing	Yes	1474 (46.78%)	745 (2.43%)	*p* < 0.001	0 (0%)
pO2 - TWMD (kPa)		-4.52 (-6.5 - -0.29) [-41.17–7.92]	-0.02 (-1.14–1.21) [-32.3–57.55]	*p* < 0.001	702 (2.08%)
SO2 - TWMD (%)		-18.1 (-28.3 - -2.01) [-84.08–56.54]	1 (-0.42–3.22) [-90.11–75.5]	*p* < 0.001	702 (2.08%)
p50 - TWMD (kPa)		0.18 (0–0.42) [-4.06–3.3]	-0.05 (-0.17–0.07) [-4.56–6.33]	*p* < 0.001	2241 (6.63%)
SpO2-SO2 - diff - TWMD (%)		18 (2.28–28.11) [-38.41–68.3]	-0.82 (-3.11–0.55) [-56.85–88.64]	*p* < 0.001	2126 (6.29%)
SpO2-SO2 - diff (match) - TWMD (%)		17.6 (2.55–27.66) [-38.06–72.01]	-0.75 (-2.87–0.44) [-70.5–59.63]	*p* < 0.001	1844 (5.46%)
PF-ratio - TWMD (kPa)		-10.68 (-20.55 - -1.3) [-82.95–33.12]	0.84 (-2.62–4.78) [-76.39–240.77]	*p* < 0.001	1588 (4.7%)

Data presented as mean (+/-SD) [range], median (IQR) [range], or count (%), as appropriate. See supplementary table 2 for full list of candidate features

**Fig. 1:**
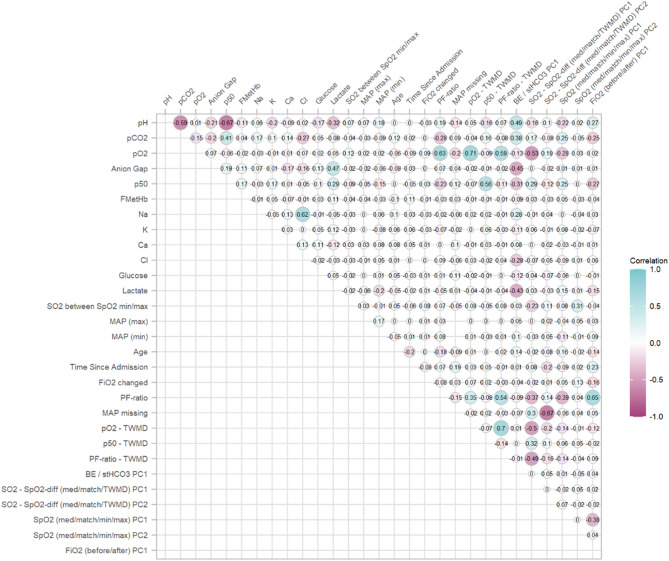
Correlation matrix of the features after reduction of dimensionality with PC of correlated features, the numbers indicate the pairwise Pearson correlation

**Fig. 2:**
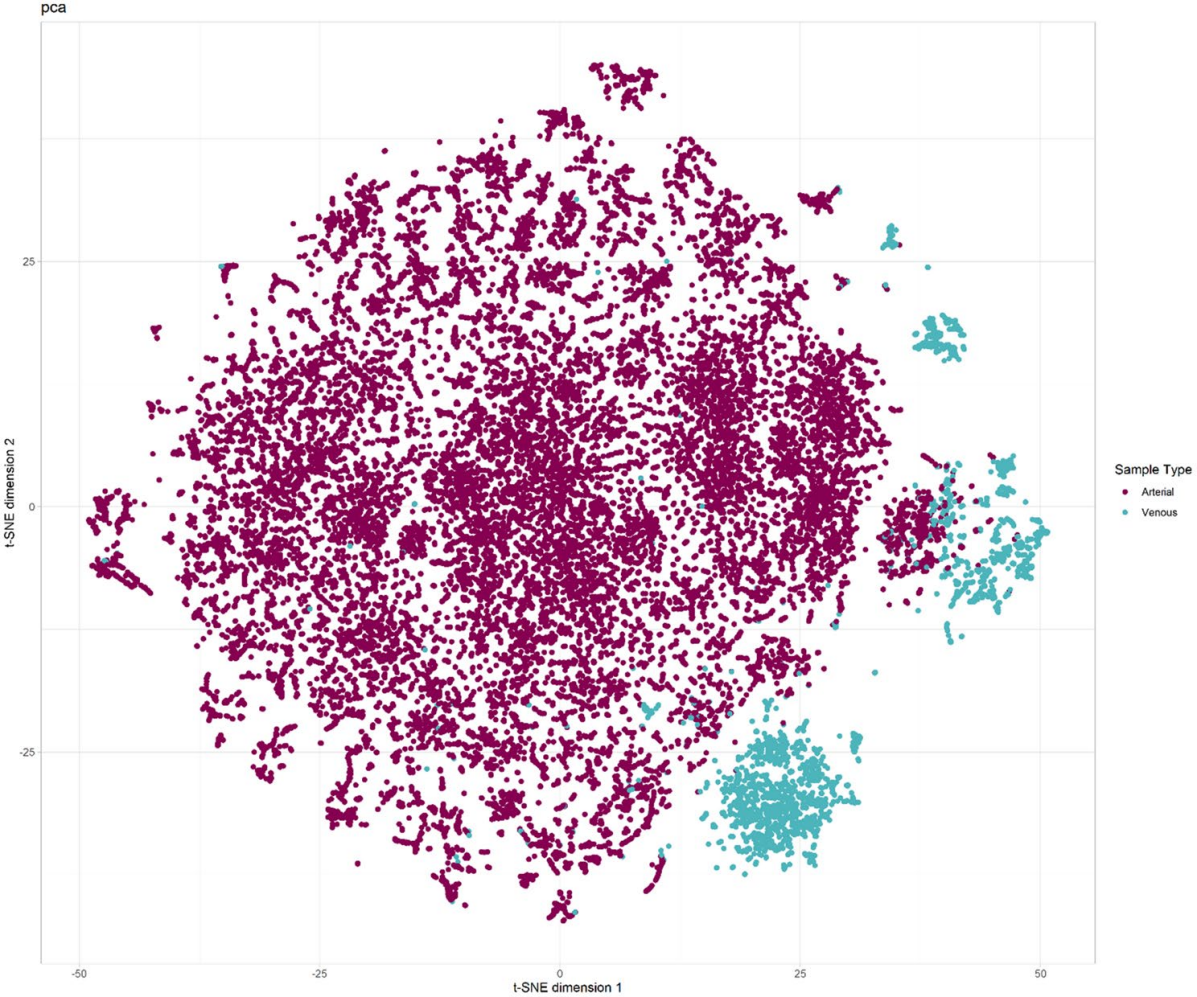
t-SNE plot from the development cohort after the dimensionality reduction process, colored by true sample class

The number of variables were reduced from 39 predictors to 29 using the PCs of correlated variables. All Pearson correlations were lower than 0.75 after the variable reduction step (Fig. 1). t-SNE of the classes in this reduced feature space still revealed a clear grouping of most venous samples (Fig. 2).

The development cohort was split proportionally by class 80%/20% into training (21589 samples) and testing (5397). Based on AUCPR in the testing set, the best-performing algorithms after grid search of the hyperparameter space were XGBoost and RF. All algorithms evaluated had an AUCPR greater than 0.987 (supplementary Table 5). The XGboost and RF algorithms were chosen for feature selection and Bayesian Optimization of hyperparameters.

In the forward stepwise selection process, the matched SpO_2_ – SO_2_ difference was the best univariate predictor for both RF and XGBoost, (Table 3). In the Bayesian optimization step in the training set, no further improvement was seen in AUCPR in the test set after Bayesian optimization of models with more than 9 features (XGBoost) and 8 features (RF), respectively (Table 3, supplementary Tables 6, 7).

**Table 3:** Results of forward stepwise feature selection with CV AUCPR after bayesian optimization at each number of features

	Random Forest		XGBoost	
Number of features	Feature	AUCPR (CV)	Feature	AUCPR (CV)
1	SpO2 - SO2-diff (match)	0.9640	SpO2 - SO2-diff (match)	0.9680
2	MAP missing	0.9847	MAP (max)	0.9838
3	pO2	0.9936	SO2	0.9934
4	SpO2 (match)	0.9957	FiO2 after	0.9964
5	Age	0.9969	PF-ratio - TWMD	0.9973
6	Hct	0.9977	Age	0.9978
7	SpO2-SO2 - diff - TWMD	0.9979	SpO2 (med)	0.9981
8	FiO2 after	0.9982	Time since admission	0.9983
9	-		SpO2-SO2 - diff (match) - TWMD	0.9984

### Holdout set results

**Fig. 3:**
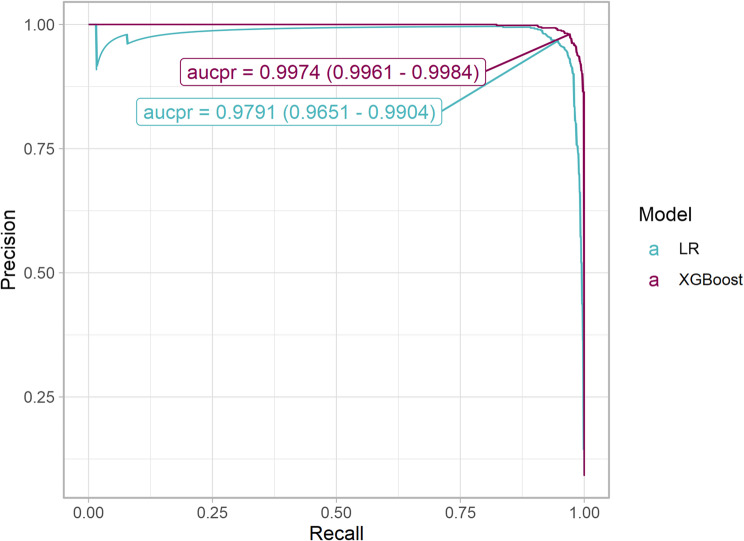
PR curves for the final XGBoost model and the Logistic Regression model in the holdout set. The point indicates the best cutoff according to maximum F1-score

**Fig. 4:**
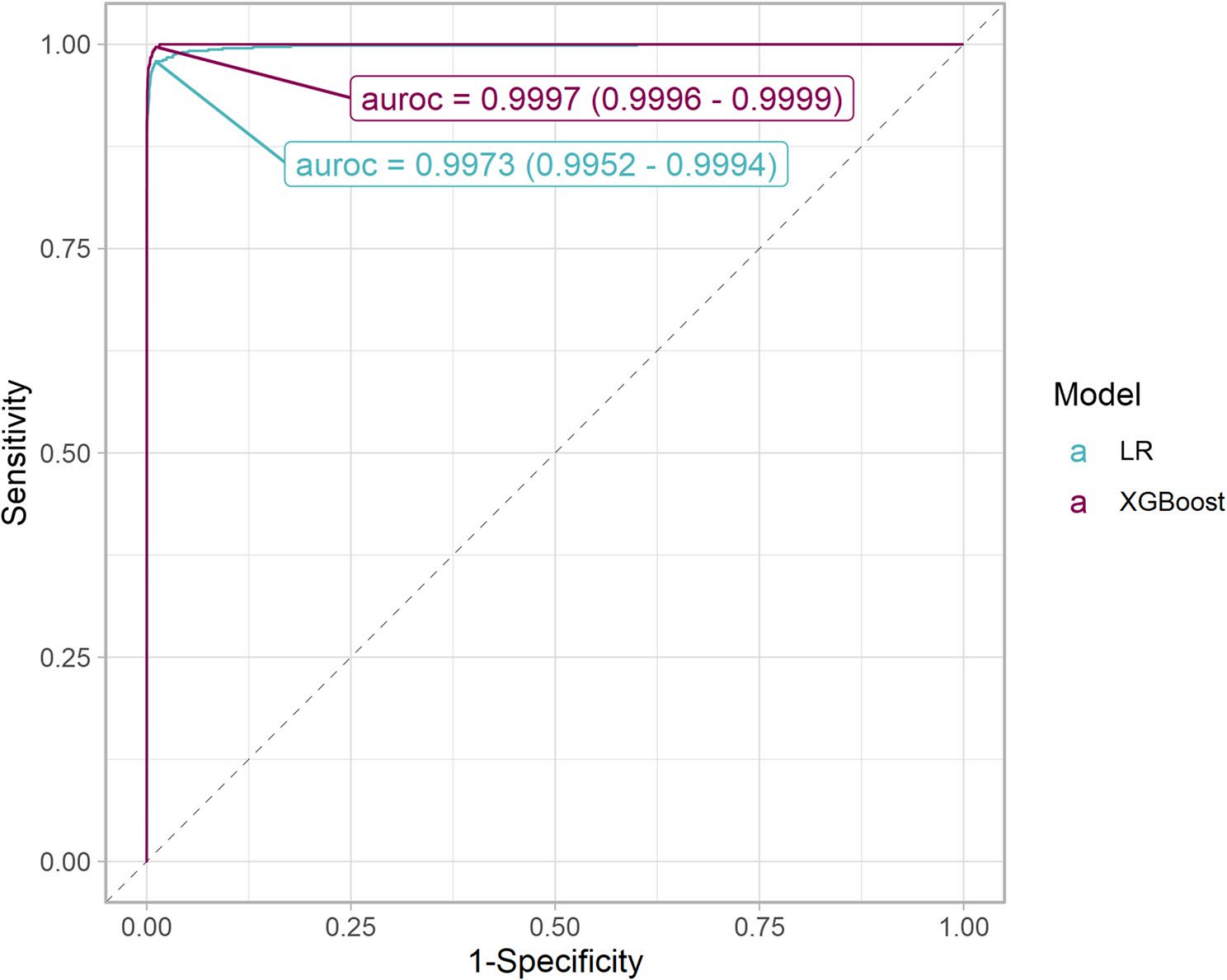
ROC curves for the final XGBoost model and the Logistic Regression model in the holdout set. The points indicate the best cutoff according to Youden’s criterion

In the holdout set, the best-performing model by AUCPR was XGBoost after Bayesian optimization with AUCPR of 0.9974 (95% CI 0.9961–0.9984, Fig. 3) and AUROC 0.9997 (95% CI 0.9996–0.9999, Fig. 4) and Brier score 0.0041 (95% CI 0.0031–0.0051). XGBoost demonstrated the best performance across all evaluated metrics except specificity, where Random Forest showed marginally better performance (Table 4). The XGboost model had significantly better discrimination than a logistic regression model (Fig. 4, *p* = 0.02).

**Table 4:** Results in the holdout set of all models after training on the full training set, ranked by AUCPR

Algorithm	AUCPR	AUROC	Accuracy	Balanced accuracy	Sensitivity	Specificity	Brier Score
XGBoost (Bayesian Optimization)	0.9974 (0.9961–0.9984)	0.9997 (0.9996–0.9999)	0.995 (0.993–0.9965)	0.9787 (0.9719–0.9849)	0.9586 (0.943–0.9742)	0.9987 (0.9978–0.9996)	0.0041 (0.0031–0.0051)
Random Forest (Bayesian Optimization)	0.9973 (0.996–0.9983)	0.9997 (0.9996–0.9999)	0.9947 (0.9927–0.9963)	0.9749 (0.9676–0.9817)	0.9506 (0.9337–0.9676)	0.9992 (0.9985–0.9999)	0.0044 (0.0035–0.0052)
XGBoost	0.9971 (0.9958–0.9982)	0.9997 (0.9995–0.9999)	0.9949 (0.9929–0.9964)	0.9764 (0.9693–0.983)	0.9538 (0.9374–0.9702)	0.999 (0.9983–0.9998)	0.0042 (0.0031–0.0054)
Random Forest	0.9966 (0.9951–0.9979)	0.9996 (0.9994–0.9998)	0.994 (0.9918–0.9957)	0.9745 (0.9671–0.9815)	0.9506 (0.9337–0.9676)	0.9984 (0.9974–0.9994)	0.0048 (0.004–0.0056)
Support Vector Machine	0.9935 (0.9904–0.9962)	0.999 (0.9983–0.9997)	0.9941 (0.992–0.9958)	0.9746 (0.9673–0.9815)	0.9506 (0.9337–0.9676)	0.9985 (0.9976–0.9995)	0.0047 (0.0036–0.0058)
Neural Network	0.993 (0.9885–0.9966)	0.9993 (0.9989–0.9997)	0.9934 (0.9912–0.9952)	0.972 (0.9644–0.9793)	0.9459 (0.9282–0.9636)	0.9982 (0.9972–0.9993)	0.0049 (0.0038–0.006)
kNN	0.9846 (0.979–0.9894)	0.9955 (0.9923–0.9988)	0.9864 (0.9833–0.989)	0.9324 (0.921–0.9433)	0.8662 (0.8396–0.8929)	0.9985 (0.9976–0.9995)	0.0095 (0.0081–0.011)
Logistic Regression	0.9791 (0.9648–0.9905)	0.9973 (0.9952–0.9994)	0.9915 (0.989–0.9935)	0.9681 (0.9602–0.9757)	0.9395 (0.9208–0.9581)	0.9968 (0.9954–0.9982)	0.0063 (0.0051–0.0077)
Regularized Linear Discriminal Analysis	0.9682 (0.9498–0.9856)	0.9977 (0.9968–0.9986)	0.9831 (0.9798–0.986)	0.9134 (0.9009–0.9259)	0.828 (0.7985–0.8575)	0.9989 (0.998–0.9997)	0.0138 (0.0118–0.016)
SpO2 - SO2 difference (matched)	0.9311 (0.911–0.9498)	0.9825 (0.9761–0.9888)	0.9871 (0.9841–0.9896)	0.9342 (0.9228–0.9451)	0.8694 (0.8431–0.8958)	0.999 (0.9983–0.9998)	0.0124 (0.0103–0.0145)

**Fig. 5:**
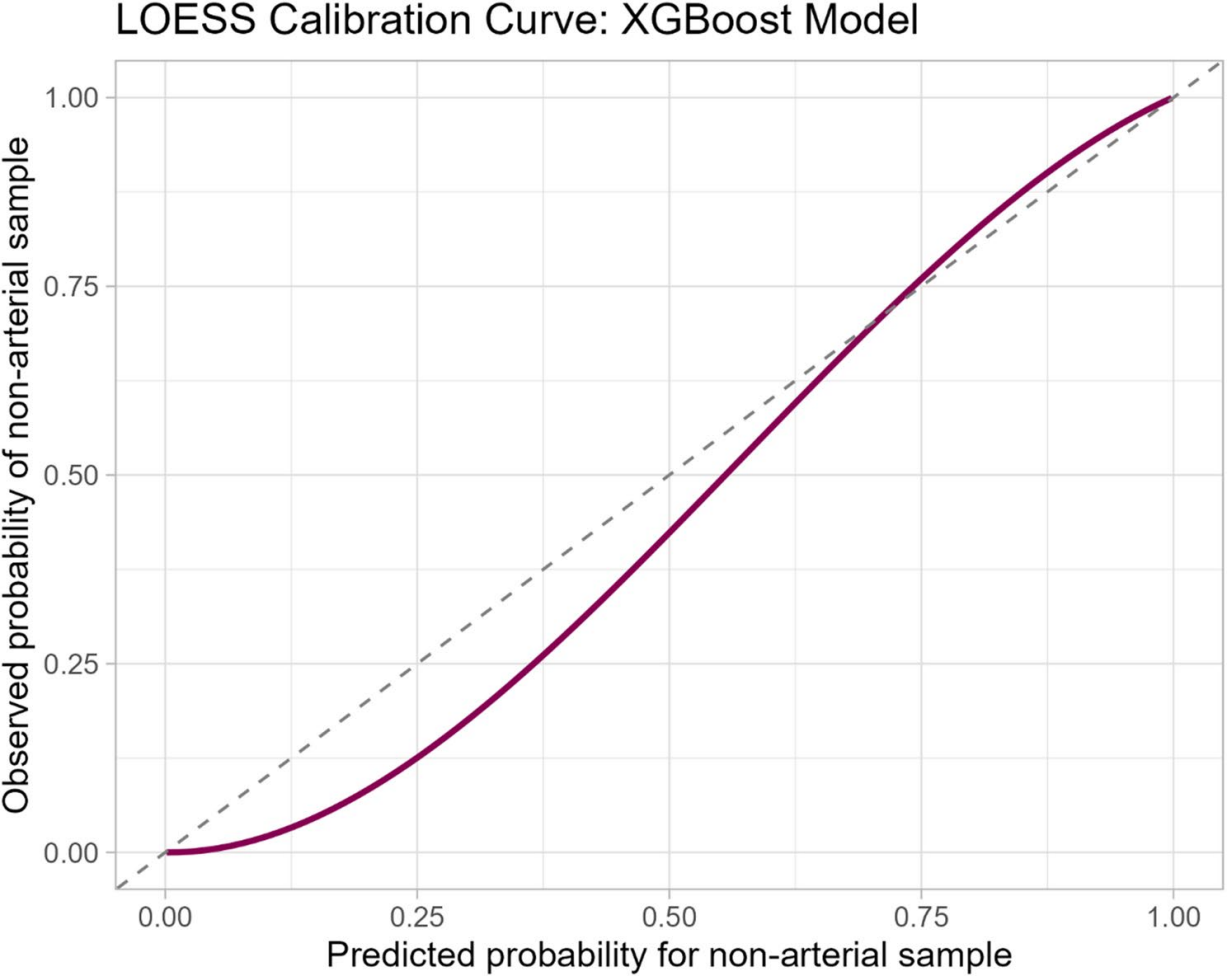
LOESS calibration plot for the XGBoost model. Abbreviations: LOESS Locally Estimated Scatterplot Smoothing, XGBoost eXtreme Gradient Boosting

Calibration assessment using logistic regression showed borderline significant underprediction of the true probabilities of non-arterial sample type overall (intercept = 0.31, *p* = 0.05), with mild underconfidence in predictions (slope 1.24, *p* < 0.01). This pattern was generally consistent with visual assessment of the calibration plot, which also revealed some underprediction in the lower range of probabilities (Fig. 5).

**Fig. 6:**
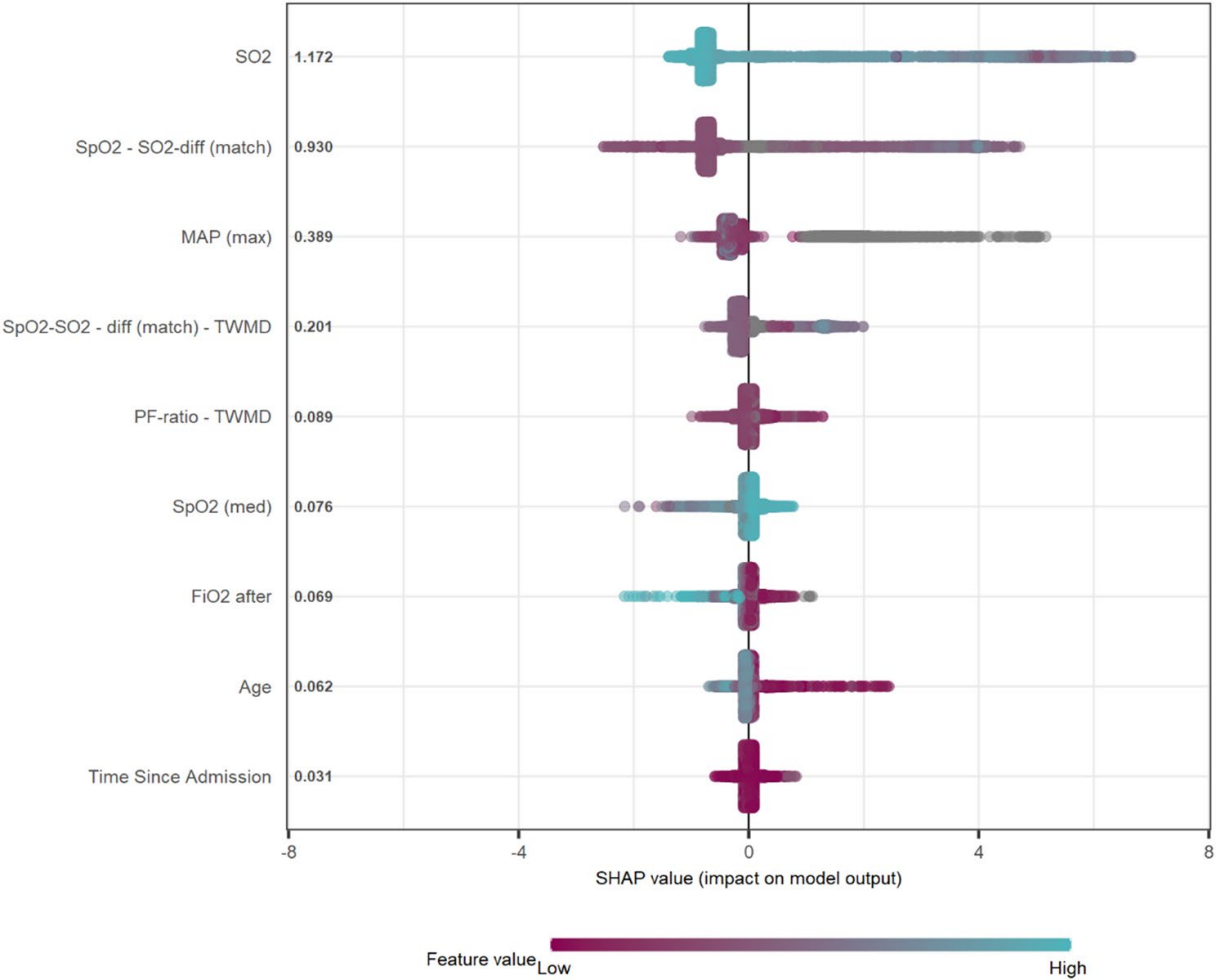
SHAP plot for the final XGBoost model. High model output corresponds to high probability of venous blood gas

Visual inspection of the SHAP plot for the optimized XGBoost model supported clinically relevant associations, for example a low SO2 and a large SpO2-SO2 difference were both strongly associated with an increased probability of non-arterial sample type (Fig. 6).

Among the blood gases in the holdout data that were initially entered into the dataset as arterial by the bedside clinician, 13 were venous according to the human rater. In this subset of the data, the XGBoost model was able to correctly identify all mislabeled blood gas samples using the cutoff with the highest F_β_ score in the test set, with 4 arterial samples misclassified as venous, corresponding to an accuracy of 99.94% (Table 5). The best accuracy in this subset of blood gases were achieved by the SVM, RF and XGboost models using the full feature set, all of them had only 1 prediction error (supplementary Table 8). For details regarding misclassifications, see Supplements Sect. 5 and Table 9.

**Table 5:** Confusion matrices for the XGBoost and logistic regression models among blood gas samples originally entered as ‘arterial’ in the holdout set

XGBoost			Logistic Regression		
True class	Predicted class		True class	Predicted class	
	Venous	Arterial		Venous	Arterial
Venous	13	0	Venous	13	0
Arterial	4	6178	Arterial	12	6170

## Discussion

### Summary of findings

We developed and validated a supervised machine learning algorithm capable of classifying blood gas samples from a mixed adult and pediatric ICU population with performance comparable to expert clinical review. In addition, we estimated the prevalence of mislabeled blood gas samples in the ICU and provided descriptive statistics for biochemical parameters based on over 30,000 manually classified samples – representing one of the largest curated blood gas datasets in the literature. Despite existing safeguards, we observed a misclassification rate of 0.44%, suggesting that sample type errors may be more common in the ICU than in standard clinical chemistry settings [2, 3, 7].

### Comparison with existing literature

The literature on blood gas sample type errors is limited, but the issue has been recognized in the context of automated SOFA score calculation, and various mitigation strategies have been proposed. For example, the Amsterdam University Medical Centers Database (AmsterdamUMCdb) SOFA algorithm regards all samples with PaO2 < 50mmHg (6.66 kPa) as non-arterial [15]. Our findings demonstrate that this threshold falls within the observed range for arterial blood gas samples from critically ill patients, highlighting the risk of misclassification when relying solely on absolute PaO2 values.

Descriptive statistics on blood gas ranges from critically ill pediatric patients are not well documented in previous research. Furthermore, while supervised machine learning has been applied to detect other sample type errors in clinical chemistry, no prior study has evaluated its use for classifying blood gas samples in the ICU setting [2]. Compared to other applications of machine learning in clinical medicine, our manual feature engineering including time-weighted averages captures trends in parameters, allowing models to account for changes in physiology.

### Strengths and limitations

Our study has strengths. We conducted a thorough manual review of all included blood gas samples to establish ground truth, independent of any modeling. The holdout set was kept entirely separate with no patient overlap, supporting the external validity of our findings and approximating prospective deployment. The large dataset enabled robust model evaluation with narrow confidence intervals and permitted subgroup-level descriptive statistics.

The final XGBoost model demonstrated high precision in detecting non-arterial samples, allowing reliable performance even in settings where the prior probability of arterial sampling is high. Calibration assessment showed a slight underprediction of non-arterial sample type, with no evidence of overfitting, supported by a low Brier score and strong performance across all metrics in the holdout set. The model was able to achieve this performance with only 9 features, and the ensemble based algorithms (XGBoost and Random Forest) were consistently better than other methods such as Logistic Regression, Support Vector Machines and Neural Networks, suggesting complex relationships between input variables and sample type. The SHAP plot suggests clinically relevant relationships, for example a high difference between SpO2 and PO2 or the absence of arterial blood pressure increases the probability of venous sample.

Our study has limitations. It is a single-center study, which may limit generalizability to other populations, institutions, or care settings. In particular, the high severity of illness (mean SAPS3 score of 60) suggests a severely ill population, where abnormal blood gas values may be more prevalent. Ground truth classification relied on retrospective review of EHR and PDMS data; reviewers lacked access to real-time bedside context, which may have influenced some classifications. Finally, the model was trained only to distinguish arterial from non-arterial samples and did not differentiate between venous subtypes, which may be relevant in certain contexts.

### Clinical implications and future research

This study demonstrates that blood gas sample type errors in the ICU can be detected automatically using machine learning, with potential for real-time implementation to improve diagnostic accuracy and enhance patient safety. Accounting for the broader physiological ranges seen in critically ill patients may reduce misclassification in automated scoring systems and improve interpretation of blood gas parameters.

The matched difference between SpO2 and SO2 was the strongest univariate predictor of sample type, outperforming traditional metrics like pO2 or the difference between SO2 and the local median SpO2. Importantly, a positive SpO2-SO2 difference was occasionally observed even in confirmed arterial samples, supporting the presence of silent hypoxemia in the ICU. Although the underlying mechanisms were not investigated in this study, they may reflect true physiological divergence due to peripheral vasoconstriction, microcirculatory failure, or elevated oxygen extraction. Future research should investigate whether this discrepancy is associated with clinical outcomes. Our model could facilitate such research in large retrospective datasets by distinguishing true silent hypoxemia from apparent SpO2-SO2 mismatches due to sample type misclassification.

Our dataset contains a mixed population of ICU patients, but the sample size is insufficient to allow meaningful subgroup analysis in specific diagnosis, such as septic shock, ARDS or cardiogenic shock. Future studies could aim to validate this model specifically in these patient groups, where severe physiological derangement could be more prevalent. Our data suggest that sample type errors may be more prevalent in patients with more severe illness, although this was a post-hoc finding and needs to be validated prospectively.

The primary motivation for developing this algorithm was to improve the accuracy of automated SOFA calculation and support sepsis recognition tools. The trained XGBoost model is available upon request, allowing other researchers to apply or validate it in retrospective datasets from other institutions, or in public ICU databases. The approach could also be adapted to identify sample type errors in other clinical settings.

Finally, our results suggest that sample type errors might be more frequent in ICU blood gas analysis than in standard clinical chemistry settings. Additional studies are needed to validate this finding in other institutions and investigate contributing factors such as staffing and workload.

## Conclusion

We developed and validated a machine learning model that classifies blood gas sample type in ICU patients using routinely collected clinical data. The model’s high precision supports its potential use in improving automated illness severity assessment and enhancing data quality in both clinical care and research. Our findings also provide new insight into the prevalence of sample type error and the characteristics of arterial and non-arterial blood gas parameters from critically ill adults and children.

## Electronic supplementary material

Below is the link to the electronic supplementary material.

Supplementary Material 1

## Data Availability

The data that support the findings of this study are not openly available due to reasons of sensitivity and are available from the corresponding author upon reasonable request. Data are located in controlled access data storage at Karolinska Institutet. Example from: 10.1186/s12910-022-00758-z.

## References

[1] Singer M, Deutschman CS, Seymour CW, et al. The third international consensus definitions for Sepsis and septic shock (Sepsis-3). JAMA. 2016;315(8):801–10. 10.1001/jama.2016.0287. 26903338 10.1001/jama.2016.0287 PMC4968574

[2] Sanchez-Pinto LN, Bennett TD, DeWitt PE et al. Development and validation of the phoenix criteria for pediatric sepsis and septic shock. Jama. Jan 21. 2024. 10.1001/jama.2024.0196 10.1001/jama.2024.0196 PMC10900964 38245897

[3] Valik JK, Ward L, Tanushi H, et al. Validation of automated sepsis surveillance based on the Sepsis-3 clinical criteria against physician record review in a general hospital population: observational study using electronic health records data. BMJ Qual Saf. 2020;09(9):735–45. 10.1136/bmjqs-2019-010123. 10.1136/bmjqs-2019-010123 PMC7467502 32029574

[4] Aakre C, Franco PM, Ferreyra M, Kitson J, Li M, Herasevich V. Prospective validation of a near real-time EHR-integrated automated SOFA score calculator. Int J Med Inf. 2017;07:103:1–6. 10.1016/j.ijmedinf.2017.04.001. 10.1016/j.ijmedinf.2017.04.001 28550994

[5] Ackermann K, Baker J, Green M, et al. Computerized clinical decision support systems for the early detection of Sepsis among adult inpatients: scoping review. J Med Internet Res Feb. 2022;23(2):e31083. 10.2196/31083. 10.2196/31083 PMC8908200 35195528

[6] Harrison AM, Yadav H, Pickering BW, Cartin-Ceba R, Herasevich V. Validation of computerized automatic calculation of the sequential organ failure assessment score. Crit Care Res Pract. 2013;2013:975672. 10.1155/2013/975672. 23936639 10.1155/2013/975672 PMC3722890

[7] Donchin Y, Gopher D, Olin M et al. A look into the nature and causes of human errors in the intensive care unit. 1995. Qual Saf Health Care. Apr 2003;12(2):143-7, discussion 147-8. 10.1136/qhc.12.2.143 10.1136/qhc.12.2.143 PMC1743697 12679512

[8] Neuraz A, Guérin C, Payet C, et al. Patient mortality is associated with staff resources and workload in the ICU: A multicenter observational study. Crit Care Med Aug. 2015;43(8):1587–94. 10.1097/ccm.0000000000001015. 10.1097/CCM.0000000000001015 25867907

[9] Bracco D, Favre JB, Bissonnette B, et al. Human errors in a multidisciplinary intensive care unit: a 1-year prospective study. Intensive Care Med. Jan 2001;27(1):137–45. 10.1007/s001340000751. 10.1007/s001340000751 11280625

[10] Vincent JL, Moreno R, Takala J, et al. The SOFA (Sepsis-related organ failure Assessment) score to describe organ dysfunction/failure. On behalf of the working group on Sepsis-Related problems of the European society of intensive care medicine. Intensive Care Med Jul. 1996;22(7):707–10. 10.1007/bf01709751. 10.1007/BF01709751 8844239

[11] Ranieri VM, Rubenfeld GD, Thompson BT, et al. Acute respiratory distress syndrome: the Berlin definition. Jama Jun. 2012;20(23):2526–33. 10.1001/jama.2012.5669. 10.1001/jama.2012.5669 22797452

[12] Fawzy A, Wu TD, Wang K, et al. Clinical outcomes associated with overestimation of oxygen saturation by pulse oximetry in patients hospitalized with COVID-19. JAMA Netw Open Aug. 2023;1(8):e2330856. 10.1001/jamanetworkopen.2023.30856. 10.1001/jamanetworkopen.2023.30856 PMC10450566 37615985

[13] Guo L, Jin Z, Gan TJ, Wang E. Silent hypoxemia in patients with COVID-19 pneumonia: A review. Med Sci Monit Oct. 2021;12:27:e930776. 10.12659/MSM.930776. 10.12659/MSM.930776 PMC8518510 34635632

[14] Kallet RH, Branson RD, Lipnick MS. Respiratory drive, dyspnea, and silent hypoxemia: A physiological review in the context of COVID-19. Respir Care Oct. 2022;67(10):1343–60. 10.4187/respcare.10075. 10.4187/respcare.10075 35501129

[15] Hastie T, Tibshirani R, Friedman JH. The elements of statistical learning: data mining, inference, and prediction. Springer; 2009.

[16] Severinghaus JW. Simple, accurate equations for human blood O2 dissociation computations. J Appl Physiol Respir Environ Exerc Physiol. Mar 1979;46(3):599–602. 10.1152/jappl.1979.46.3.599. 10.1152/jappl.1979.46.3.599 35496

[17] Siggaard-Andersen O. An acid-base chart for arterial blood with normal and pathophysiological reference areas. Scand J Clin Lab Invest May. 1971;27(3):239–45. 10.3109/00365517109080214. 10.3109/00365517109080214 5581186

[18] Brent RP. Algorithms for minimization without derivatives. Prentice-Hall; 1973: Chap. 4: an algorithm with guaranteed convergence for finding a zero of a function.

[19] van der Maaten L, Hinton GE. Visualizing High-Dimensional data using t-SNE. J Mach Learn Res. 2008;9:2579–605.

[20] Breiman L, Random, Forests. Machine Learning. 2001/10/01. 2001;45(1):5–32. 10.1023/A:1010933404324

[21] Chen T, Guestrin C, XGBoost:. A scalable tree boosting system. Presented at: Proceedings of the 22nd ACM SIGKDD International Conference on Knowledge Discovery and Data Mining; 2016; San Francisco, California, USA. 10.1145/2939672.2939785

[22] Cortes C, Vapnik V. Support-vector networks. Machine Learning. 1995/09/01. 1995;20(3):273–297. 10.1007/BF00994018

[23] Schmidhuber J. Deep learning in neural networks: an overview. Neural Networks. 2015/01/01. 2015;61:85–117. 10.1016/j.neunet.2014.09.003 10.1016/j.neunet.2014.09.003 25462637

[24] Guo Y, Hastie T, Tibshirani R. Regularized linear discriminant analysis and its application in microarrays. Biostatistics. 2006;8(1):86–100. 10.1093/biostatistics/kxj035 16603682 10.1093/biostatistics/kxj035

[25] DeLong ER, DeLong DM, Clarke-Pearson DL. Comparing the areas under two or more correlated receiver operating characteristic curves: a nonparametric approach. Biometrics. 1988:837–45. 3203132

[26] Lundberg SM, Lee S-I. A unified approach to interpreting model predictions. presented at: Proceedings of the 31st International Conference on Neural Information Processing Systems; 2017; Long Beach, California, USA.

[27] Thoral PJ, Peppink JM, Driessen RH, Sharing ICU Patient Data Responsibly Under the Society of Critical Care Medicine/European Society of Intensive Care Medicine Joint Data Science Collaboration, et al. The Amsterdam university medical centers database (AmsterdamUMCdb) example. Crit Care Med Jun. 2021;1(6):e563–77. 10.1097/ccm.0000000000004916. 10.1097/CCM.0000000000004916 PMC8132908 33625129

