# Supplementary material for: Beyond labels: determining the true type of blood gas samples in ICU patients through supervised machine learning
Source: BMC Med Inform Decis Mak. 2025 Jul 24;25:275. doi: 10.1186/s12911-025-03115-3 (PMC12288198; doi:10.1186/s12911-025-03115-3)
Supplement: Supplementary file 1 — Supplementary Material 1 [file 12911_2025_3115_MOESM1_ESM.docx]

# Supplementary material

Table of Contents

[Supplementary material 1](#_Toc201224078)

[1. Variables 1](#_Toc201224079)

[1.1. Variable definitions 1](#_Toc201224080)

[1.2 Variable ranges in the full cohort, and univariate 5-fold-crossvalidated AUROC for sample type in the development cohort. 5](#_Toc201224081)

[1.3 Blood gas parameter ranges in the full cohort, by patient age group and sample type 9](#_Toc201224082)

[2. Formulae used for calculations of missing values 10](#_Toc201224083)

[3. Models, libraries and training procedures 11](#_Toc201224084)

[3.1. Grid search procedure and results 11](#_Toc201224085)

[3.2. Forward stepwise feature selection 12](#_Toc201224086)

[3.3. Parameter space during Bayesian optimization phase 12](#_Toc201224087)

[4. Results from Bayesian optimization 13](#_Toc201224088)

[Random forest 13](#_Toc201224089)

[Xgboost 13](#_Toc201224090)

[5. Summary of missclassifications from XGBoost model 14](#_Toc201224091)

[6. Confusion matrices 14](#_Toc201224092)

[7. Manual review 16](#_Toc201224093)

[8. References 16](#_Toc201224094)

## Variables

### Variable definitions

| Variable | Description | Unit | Definition |
| --- | --- | --- | --- |
| pH | pH | - | pH from blood gas sample |
| pCO2 | Partial pressure of Carbon Dioxide | kPa | pCO2 from blood gas sample |
| pO2 | Partial pressure of oxygen | kPa | pO2 from blood gas sapmle |
| BE | Base Excess | mmol/L | Base Excess from blood gas sample |
| stHCO3 | Standard Bicarbonate | mmol/L | Standard Bicarbonate from blood gas sample |
| Anion Gap | Anion gap | mmol/L | Anion gap from blood gas sample |
| SO2 | Saturation of hemoglobin | % | From blood gas sample |
| p50 | Partial pressure of oxygen for 50% saturation of hemoglobin | kPa | From blood gas sample |
| FMetHb | Methhemoglobin % | % | From blood gas sample |
| Hb | Hemoglobin level | g/L | From blood gas sample |
| Hct | Hematocrit | % | From blood gas sample |
| Na | Plasma Sodium | mmol/L | From blood gas sample |
| K | Plasma Potassium | mmol/L | From blood gas sample |
| Ca | Plasma ionized calcium | mmol/L | From blood gas sample |
| Cl | Plasma cloride | mmol/L | From blood gas sample |
| Glucose | Plasma glucose | mmol/L | From blood gas sample |
| Lactate | Plasma lactate | mmol/L | From blood gas sample |
| SpO2 (med) | Median of peripheral saturation (SpO2) at sample time | % | Median value of all SpO2 values in the EHR during 900 seconds prior to the blood gas analysis. *Rationale : robust measurement of actual SpO2 value. The timeframe of 900 seconds prior is chosen as the actual sampling time is unknown, but likely a few minutes prior to the time of analysis.* |
| SpO2 (match) | Best matched SpO2 | % | Closest matching SpO2 value to SO2 value of blood gas. Best match defined according to the following function, for each SpO2i in the set of SpO2 values within +/- 600 seconds from blood gas sampling time: $\underset{i}{which.\min} \left\vert SO2- {SpO2}_{i} \right\vert+\frac{1}{14400}{(SO2\_sampletime-SpO2\_sampletime_{i})}^{2}$. *Rationale: The true time of blood gas sample is not known, and could vary slightly from analysis. The ‘best matching’ SpO2 should preferably be close in time to the time of analysis, thus the quadratic penalty.* |
| SpO2 (min) | Lowest SpO2 value | % | The lowest SpO2 value +/- 600 seconds from blood gas sample time.  Rationale: *The range of SpO2 values around sample time gives an idea of what SO2 to expect.* |
| SpO2 (max) | Highest SpO2 value | % | The highest SpO2 value +/- 600 seconds from blood gas sampling  Rationale: *The range of SpO2 values around sample time gives an idea of what SO2 to expect.* |
| SpO2 - SO2-diff (med) | Difference between median SpO2 and SO2 | % | SpO2 (med) – SO2  Rationale: *From physiology, this entity should be close to 0 for arterial samples, but high for venous samples. It is not known if the robust estimate (median) or the closest estimate (match) is the best estimate of the SpO2.* |
| SpO2 - SO2-diff (match) | Difference between best matching SpO2 and SO2 | % | SpO2 (match)– SO2  Rationale: *From physiology, this entity should be close to 0 for arterial samples, but high for venous samples. It is not known if the robust estimate (median) or the closest estimate (match) is the best estimate of the SpO2.* |
| SO2 between SpO2 min/max | SO2 between lowest and highest SpO2 | - | 1 if SpO2 (min) ≤ SO2 ≤ SpO2 (max), 0 otherwise  Rationale: *the SaO2 is likely to be higher than SpO2-min, and should approximate SpO2(max). Thus, this entity is more likely to be true for arterial samples.* |
| MAP (max) | Maximum of mean arterial pressure (MAP) | mmHg | Highest MAP value recorded between +/- 600 seconds from blood gas sampling  Rationale: *High MAP values could represent flushing artifacts after blood gas sampling, corresponding to higher likelihood of arterial sample.* |
| MAP (min) | Minimum of mean arterial pressure | mmHg | Lowest MAP value recorded between +/- 600 seconds from blood gas sampling  Rationale: *With very low MAP values, the patient might be critically unstable and thus the normal relationship between SpO2 and SO2 may differ – thus this entity might add information in certain cases.* |
| Age | Age at admission | Years | Age at ICU admission  Rationale: *HIgh venous SO2 is more likely seen in young, healthy patients, than in the elderly – this entity might add information in certain cases.* |
| time_since_admission | Time since admission | Seconds | Time since ICU admission  Rationale: *The presence of absence of an arterial line might be somehow related to the length of stay, being more likely to be present in the earlier phase of ICU care.* |
| FiO2 before | Fraction of inspired oxygen (FiO2) before blood gas sample | % | Last recorded FiO2 value at least 300 seconds before blood gas sampling time |
| FiO2 after | FIO2 after blood gas sample | % | First recorded FiO2 value at least 600 seconds after blood gas sampling time |
| FiO2 changed | Change in FiO2 before and after blood gas | - | 1 if round $\left( \frac{{FiO2}_{before}}{5} \right)= \mathrm{round}\left( \frac{{FiO2}_{after}}{5} \right)$, 0 otherwise |
| PF-ratio | P/F Ratio | kPa | Defined as PaO2 / FiO2_before_ with PaO2 in kPa and FiO2 between 0-1 instead of 0-100. |
| MAP missing | No MAP Values recorded | - | 1 if no MAP values recorded between +/- 600 seconds from sample time, 0 otherwise |
| pO2 - TWMD | Difference between current pO2 and time weighted mean of pO2. | kPa | pO2-TWM = Tri-cubic time-weighted-mean of pO2 from -24 hours of sampling, to sample time. Each value weighted by Weight_dt_ when calculating the mean, where dt is the time difference of each previous sample in that time range to the current sample, scaled in a range where 0 seconds correspond to 0 and 24 hours correspond to 1 and  ${Weight}_{dt}= \left( 1-{dt}^{3} \right)^{3}$ . Time Weighted Mean (TWM) of PO2 (pO2 – TWM) calculated as the inner product of all pO2s and their corresponding weights, divided by the sum of all weights. Time weighted mean difference (TWMD) calculated as : pO2-TMWD = pO2 – PO2-TWM.  Rationale: *the time weighted mean will represent this patients ‘trend’ in pO2 over time and integrate many blood gas samples. Since the sample frequency for arterial blood is generally higher than for venous blood, venous blood gas samples are more likely to differ from this long term trend. Note, this is performed in all samples, not only those marked ‘arterial’, since we purposely removed the given label in all feature engineering.* |
| SO2 - TWMD | Difference between current sO2 and time weighted mean of sO2. | % | See pO2-TMWD, but with SO2 instead of pO2. |
| P50 - TWMD | Difference between current p50 and time weighted mean of p50. | kPa | See pO2-TMWD, but with p50 instead of pO2. |
| SpO2-SO2 - diff - TWMD | Difference between current SpO2 - SO2-diff (med) and time weighted mean of SpO2 - SO2-diff (med) | % | See pO2-TMWD, but with SpO2-SO2 - diff (med) instead of pO2. |
| SpO2-SO2 - diff (match) - TWMD | Difference between current SpO2 - SO2-diff (med)and time weighted mean of SpO2 - SO2-diff (match). | % | See pO2-TMWD, but with SpO2-SO2 - diff (match) instead of pO2. |
| PF-ratio - TWMD | Difference between current PF-ratio and time weighted average of PF-ratio. | kPa | See pO2_wtd, but with PF-ratio instead of pO2. |

Supplementary table 1: Variable definitions

### 1.2 Variable ranges in the full cohort, and univariate 5-fold-crossvalidated AUROC for sample type in the development cohort.

| Variable | Value | venous or central venous (n = 3151) | arterial (n = 30649) | p | miss | auc |
| --- | --- | --- | --- | --- | --- | --- |
| pH |  | 7.38 (7.32 - 7.42) [6.76 - 7.61] | 7.42 (7.37 - 7.46) [6.68 - 7.81] | p<0.001 | 0.6842 (0.6624 - 0.7059) | 243 (0.72%) |
| pCO2 (kPa) |  | 5.69 (5.09 - 6.63) [2.13 - 21.1] | 5.05 (4.46 - 5.84) [1.08 - 24.7] | p<0.001 | 0.6808 (0.6586 - 0.7031) | 260 (0.77%) |
| pO2 (kPa) |  | 4.98 (4.35 - 5.72) [2 - 17.2] | 10.9 (9.64 - 12.5) [1.05 - 75.8] | p<0.001 | 0.9881 (0.9813 - 0.9949) | 0 (0%) |
| BE (mmol/L) |  | -0.03 (+/- 4.72) [-27.6 - 53.11] | 0.2 (+/- 4.66) [-27.7 - 25.8] | p=0.362 | 0.5185 (0.4924 - 0.5446) | 313 (0.93%) |
| stHCO3 (mmol/L) |  | 24.1 (21.9 - 26.2) [0 - 82.5] | 24.7 (22.4 - 26.9) [0 - 50.7] | p<0.001 | 0.5532 (0.5273 - 0.5791) | 313 (0.93%) |
| Anion Gap (mmol/L) |  | 6.6 (4.9 - 8.6) [-14.2 - 38.7] | 6.5 (4.7 - 8.6) [-8.8 - 34.9] | p=1 | 0.5105 (0.4846 - 0.5363) | 878 (2.6%) |
| SO2 (%) |  | 68.8 (61.8 - 75.1) [12 - 99.2] | 96.1 (94.3 - 97.4) [3.5 - 100.1] | p<0.001 | 0.9877 (0.9807 - 0.9948) | 0 (0%) |
| p50 (kPa) |  | 3.85 (3.7 - 4) [0 - 9.19] | 3.45 (3.28 - 3.67) [2.09 - 12.12] | p<0.001 | 0.8149 (0.7999 - 0.8299) | 1542 (4.56%) |
| FMetHb (%) |  | 1.6 (1.2 - 1.8) [0 - 8.4] | 1.5 (1.1 - 1.7) [0 - 7.7] | p<0.001 | 0.5454 (0.5191 - 0.5716) | 247 (0.73%) |
| Hbg (g/L) |  | 93 (84 - 106) [5.4 - 199] | 92 (84 - 107) [10 - 203] | p=1 | 0.4964 (0.4713 - 0.5214) | 168 (0.5%) |
| Hct (%) |  | 0.29 (0.26 - 0.33) [0.03 - 0.61] | 0.29 (0.26 - 0.33) [0.04 - 0.8] | p=1 | 0.4938 (0.4686 - 0.519) | 1088 (3.22%) |
| Na (mmol/L) |  | 138 (136 - 141) [64 - 214] | 139 (136 - 142) [103 - 174] | p<0.001 | 0.542 (0.5161 - 0.5679) | 354 (1.05%) |
| K (mmol/L) |  | 4.3 (3.9 - 4.6) [1.2 - 19.7] | 4.2 (3.9 - 4.5) [0 - 14.5] | p<0.001 | 0.5277 (0.5001 - 0.5553) | 291 (0.86%) |
| Ca (mmol/L) |  | 1.16 (1.07 - 1.23) [0.23 - 2.72] | 1.15 (1.07 - 1.2) [0.68 - 2.12] | p<0.001 | 0.5338 (0.5052 - 0.5625) | 380 (1.12%) |
| Cl (mmol/L) |  | 106.49 (+/- 5.69) [70 - 136] | 108.15 (+/- 5.4) [70 - 140] | p<0.001 | 0.5896 (0.5623 - 0.6168) | 290 (0.86%) |
| Glucose (mmol/L) |  | 7.8 (6.7 - 9.4) [2.5 - 49] | 7.6 (6.6 - 9) [0.5 - 44] | p<0.001 | 0.5399 (0.5124 - 0.5675) | 130 (0.38%) |
| Lactate (mmol/L) |  | 1.5 (1.1 - 2.2) [0.2 - 29] | 1.4 (1 - 2.1) [0.2 - 29] | p<0.001 | 0.5616 (0.5366 - 0.5866) | 585 (1.73%) |
| SpO2 (med) (%) |  | 97 (95 - 99) [69 - 100] | 96 (94 - 98) [38 - 100] | p<0.001 | 0.5619 (0.5361 - 0.5876) | 1443 (4.27%) |
| SpO2 (match) (%) |  | 96 (94 - 98) [20 - 100] | 96 (94 - 98) [28 - 100] | p=0.378 | 0.4902 (0.4632 - 0.5173) | 1161 (3.43%) |
| SpO2 (min) (%) |  | 96 (93 - 97.2) [20 - 100] | 95 (93 - 97) [2 - 100] | p<0.001 | 0.5439 (0.5179 - 0.5699) | 1161 (3.43%) |
| SpO2 (max) (%) |  | 98 (96.16 - 100) [38 - 100] | 98 (96 - 99) [28 - 100] | p<0.001 | 0.5695 (0.544 - 0.5949) | 1161 (3.43%) |
| SpO2 - SO2-diff (med) (%) |  | 27.7 (21.4 - 34.4) [-6.1 - 87] | 0.5 (-0.7 - 1.7) [-50 - 88.5] | p<0.001 | 0.9897 (0.9855 - 0.9939) | 1443 (4.27%) |
| SpO2 - SO2-diff (match) (%) |  | 27 (20.5 - 33.8) [-18.7 - 88] | 0.4 (-0.5 - 1.4) [-44.9 - 66] | p<0.001 | 0.9901 (0.985 - 0.9952) | 1161 (3.43%) |
| SO2 between SpO2 min/max | No | 2988 (94.83%) | 16393 (53.49%) |  |  |  |
| SO2 between SpO2 min/max | Yes | 163 (5.17%) | 14256 (46.51%) | p<0.001 | 0.7077 (0.6962 - 0.7193) | 0 (0%) |
| MAP (max) (mmHg) |  | 82 (72 - 114) [-4 - 360] | 87 (74 - 125) [0 - 361] | p<0.001 | 0.4203 (0.3957 - 0.4448) | 2219 (6.57%) |
| MAP (min) (mmHg) |  | 66 (60 - 74) [-35 - 337] | 69 (62 - 78) [-111 - 269] | p<0.001 | 0.5144 (0.4928 - 0.5361) | 2219 (6.57%) |
| Age (Years) |  | 58 (36 - 66) [0 - 93] | 63 (49 - 69) [0 - 93] | p<0.001 | 0.5771 (0.5512 - 0.603) | 0 (0%) |
| Time Since Admission (Seconds) |  | 383940 (76680 - 1135680) [-10980 - 6720780] | 258840 (73980 - 678540) [-2996460 - 6303300] | p<0.001 | 0.5677 (0.5383 - 0.5972) | 0 (0%) |
| FiO2 before (%) |  | 30 (24.9 - 40) [19.3 - 100] | 34.7 (25 - 45.1) [18.6 - 100] | p<0.001 | 0.5602 (0.5345 - 0.5858) | 913 (2.7%) |
| FiO2 after (%) |  | 30 (24.8 - 40) [19.4 - 100] | 34.8 (25 - 45.1) [18.6 - 100] | p<0.001 | 0.5587 (0.5331 - 0.5842) | 869 (2.57%) |
| FiO2 changed | No | 2285 (72.52%) | 22450 (73.25%) |  |  |  |
| FiO2 changed | Yes | 586 (18.6%) | 6771 (22.09%) |  |  |  |
| FiO2 changed | N/A | 280 (8.89%) | 1428 (4.66%) | p<0.001 | 0.518 (0.5 - 0.536) | 1708 (5.05%) |
| PF-ratio (kPa) |  | 16.3 (11.8 - 21.02) [3.65 - 81.9] | 34 (23.21 - 44.8) [3.74 - 291.7] | p<0.001 | 0.8553 (0.8407 - 0.8699) | 913 (2.7%) |
| MAP missing | No | 1677 (53.22%) | 29904 (97.57%) |  |  |  |
| MAP missing | Yes | 1474 (46.78%) | 745 (2.43%) | p<0.001 | 0.723 (0.7012 - 0.7449) | 0 (0%) |
| pO2 - TWMD (kPa) |  | -4.52 (-6.5 - -0.29) [-41.17 - 7.92] | -0.02 (-1.14 - 1.21) [-32.3 - 57.55] | p<0.001 | 0.7881 (0.7656 - 0.8106) | 702 (2.08%) |
| SO2 - TWMD (%) |  | -18.1 (-28.3 - -2.01) [-84.08 - 56.54] | 1 (-0.42 - 3.22) [-90.11 - 75.5] | p<0.001 | 0.8352 (0.8088 - 0.8616) | 702 (2.08%) |
| p50 - TWMD (kPa) |  | 0.18 (0 - 0.42) [-4.06 - 3.3] | -0.05 (-0.17 - 0.07) [-4.56 - 6.33] | p<0.001 | 0.7348 (0.7107 - 0.759) | 2241 (6.63%) |
| SpO2-SO2 - diff - TWMD (%) |  | 18 (2.28 - 28.11) [-38.41 - 68.3] | -0.82 (-3.11 - 0.55) [-56.85 - 88.64] | p<0.001 | 0.8259 (0.7996 - 0.8521) | 2126 (6.29%) |
| SpO2-SO2 - diff (match) - TWMD (%) |  | 17.6 (2.55 - 27.66) [-38.06 - 72.01] | -0.75 (-2.87 - 0.44) [-70.5 - 59.63] | p<0.001 | 0.8295 (0.8028 - 0.8561) | 1844 (5.46%) |
| PF-ratio - TWMD (kPa) |  | -10.68 (-20.55 - -1.3) [-82.95 - 33.12] | 0.84 (-2.62 - 4.78) [-76.39 - 240.77] | p<0.001 | 0.7976 (0.7766 - 0.8186) | 1588 (4.7%) |

Supplementary table 2: Variable ranges per class in the full dataset, number of missing values, and univariate 5-fold cross-validated predictive performance for sample type in the development cohort

### 1.3 Blood gas parameter ranges in the full cohort, by patient age group and sample type

|  |  | arterial | | | | | venous | | | | |
| --- | --- | --- | --- | --- | --- | --- | --- | --- | --- | --- | --- |
| Patients | Variable | Mean | Median | SD | Range | 95% Range | Mean | Median | SD | Range | 95% Range |
| Adult | pH | 7.41 | 7.42 | 0.08 | 6.68 - 7.81 | 7.21 - 7.52 | 7.36 | 7.38 | 0.08 | 6.76 - 7.61 | 7.17 - 7.47 |
| Adult | pCO2 | 5.37 | 5.07 | 1.48 | 1.08 - 24.7 | 3.44 - 9.16 | 6.21 | 5.77 | 1.59 | 2.13 - 21.1 | 4.19 - 10.38 |
| Adult | pO2 | 11.44 | 10.8 | 3.43 | 1.05 - 70.7 | 7.61 - 18.5 | 5.14 | 4.96 | 1.29 | 2 - 17.2 | 3.26 - 8.12 |
| Adult | BE | 0.27 | 0.4 | 4.66 | -27.7 - 25.8 | -9.1 - 9.5 | 0.1 | 0.3 | 4.67 | -27.6 - 53.11 | -8.9 - 10.48 |
| Adult | stHCO3 | 24.77 | 24.7 | 4.03 | 0 - 50.7 | 17 - 33.1 | 24.18 | 24.2 | 4.1 | 0 - 82.5 | 16.9 - 33.59 |
| Adult | AnGap | 6.89 | 6.6 | 3.47 | -8.8 - 34.9 | 1 - 15 | 6.95 | 6.5 | 3.6 | -14.2 - 38.7 | 1.1 - 15 |
| Adult | SO2 | 95.37 | 96.1 | 3.34 | 3.5 - 100.1 | 87.7 - 99.1 | 67.54 | 68.3 | 11.09 | 12 - 99 | 42.2 - 86.78 |
| Adult | p50 | 3.53 | 3.45 | 0.41 | 2.09 - 12.12 | 3.02 - 4.51 | 3.88 | 3.87 | 0.29 | 0 - 5.73 | 3.41 - 4.47 |
| Adult | FMetHb | 1.44 | 1.5 | 0.57 | 0 - 7.7 | 0.5 - 2.5 | 1.51 | 1.6 | 0.58 | 0 - 8.4 | 0.6 - 2.5 |
| Adult | Hb | 97.43 | 93 | 18.99 | 10 - 203 | 72 - 143 | 96.97 | 93 | 19.56 | 5.4 - 199 | 72 - 148.2 |
| Adult | Hct | 0.3 | 0.29 | 0.06 | 0.04 - 0.8 | 0.22 - 0.44 | 0.3 | 0.29 | 0.06 | 0.03 - 0.61 | 0.22 - 0.46 |
| Adult | Na | 139.5 | 139 | 5.14 | 103 - 174 | 130 - 151 | 138.7 | 138 | 5.65 | 64 - 196 | 130 - 150 |
| Adult | K | 4.26 | 4.2 | 0.5 | 0 - 14.5 | 3.4 - 5.3 | 4.35 | 4.3 | 0.78 | 1.2 - 19.7 | 3.2 - 5.8 |
| Adult | Ca | 1.14 | 1.15 | 0.11 | 0.68 - 2.12 | 0.95 - 1.34 | 1.13 | 1.15 | 0.15 | 0.23 - 2.72 | 0.87 - 1.37 |
| Adult | Cl | 108.1 | 108 | 5.43 | 70 - 140 | 98 - 120 | 106.4 | 106 | 5.85 | 70 - 136 | 95 - 119 |
| Adult | Glucose | 8.12 | 7.6 | 2.49 | 0.5 - 44 | 4.9 - 14.36 | 8.61 | 7.9 | 3.22 | 2.5 - 40 | 4.9 - 16.78 |
| Adult | Lactate | 1.87 | 1.4 | 1.85 | 0.2 - 29 | 0.5 - 6.7 | 2.03 | 1.6 | 1.82 | 0.3 - 29 | 0.6 - 7 |
| Pediatric | pH | 7.41 | 7.42 | 0.07 | 7.06 - 7.57 | 7.23 - 7.51 | 7.39 | 7.4 | 0.07 | 6.88 - 7.56 | 7.24 - 7.48 |
| Pediatric | pCO2 | 4.84 | 4.78 | 1.04 | 1.12 - 11.7 | 3.16 - 6.84 | 5.39 | 5.12 | 1.35 | 2.43 - 13.6 | 3.61 - 9.12 |
| Pediatric | pO2 | 12.64 | 11.9 | 4.96 | 4.35 - 75.8 | 7.7 - 20.5 | 5.69 | 5.11 | 2.09 | 3.39 - 17.2 | 3.72 - 11.95 |
| Pediatric | BE | -1.33 | -0.85 | 4.44 | -25.4 - 9 | -10.5 - 5.9 | -0.83 | -0.6 | 4.94 | -23.1 - 11.6 | -12.26 - 7.67 |
| Pediatric | stHCO3 | 23.4 | 23.7 | 3.69 | 6.5 - 32.7 | 15.9 - 29.7 | 23.52 | 23.5 | 4.05 | 7 - 34.9 | 14.86 - 31.07 |
| Pediatric | AnGap | 6.31 | 6 | 2.81 | -1.5 - 22.5 | 1.5 - 12.3 | 7.2 | 6.9 | 3.43 | 0.01 - 20.9 | 0.96 - 16.54 |
| Pediatric | SO2 | 96.53 | 97.4 | 3.37 | 45.7 - 100 | 89.43 - 99.4 | 72.72 | 71.1 | 11.36 | 44 - 99.2 | 53.36 - 96.99 |
| Pediatric | p50 | 3.41 | 3.37 | 0.28 | 2.72 - 4.98 | 2.98 - 4.08 | 3.74 | 3.75 | 0.36 | 2.79 - 9.19 | 3.28 - 4.11 |
| Pediatric | FMetHb | 1.45 | 1.6 | 0.46 | 0 - 2.6 | 0.6 - 2.1 | 1.56 | 1.6 | 0.5 | 0 - 3 | 0.6 - 2.5 |
| Pediatric | Hb | 90.88 | 88 | 15.7 | 63 - 156 | 72 - 142 | 95.29 | 91 | 18.26 | 60 - 151 | 73 - 137 |
| Pediatric | Hct | 0.28 | 0.27 | 0.05 | 0.2 - 0.48 | 0.22 - 0.44 | 0.29 | 0.28 | 0.06 | 0.14 - 0.46 | 0.23 - 0.42 |
| Pediatric | Na | 137.3 | 137 | 3.36 | 126 - 150 | 132 - 146 | 139.1 | 138 | 6.4 | 122 - 214 | 132.4 - 148.6 |
| Pediatric | K | 4.11 | 4.1 | 0.51 | 1 - 8.1 | 3.2 - 5.1 | 4.13 | 4.1 | 0.55 | 2.4 - 6.6 | 3.1 - 5.3 |
| Pediatric | Ca | 1.15 | 1.16 | 0.09 | 0.82 - 1.49 | 0.94 - 1.29 | 1.21 | 1.21 | 0.09 | 0.3 - 1.41 | 1.04 - 1.35 |
| Pediatric | Cl | 108.4 | 108 | 4.62 | 95 - 121 | 99 - 118 | 107.3 | 108 | 4.48 | 87 - 121 | 98 - 115 |
| Pediatric | Glucose | 7.82 | 7.2 | 2.62 | 1.9 - 25 | 4.7 - 14.28 | 8.58 | 7 | 4.98 | 3.2 - 49 | 4.75 - 23.42 |
| Pediatric | Lactate | 1.83 | 1.4 | 1.75 | 0.3 - 17 | 0.4 - 6.86 | 1.57 | 1.3 | 1.56 | 0.2 - 19 | 0.4 - 4.6 |

Supplementary table 3 : Ranges of blood gas parameters per sample type and patient age group (Pediatric = <18 years, Adult = >= 18 years)

## Formulae used for calculations of missing values

When any blood gas parameter with a known mathematical relationship to other parameters was missing, and the required parameters were known, they were calculated using the following equations.

1. Severinghaus’ formula for fractional saturation (S) as function of P_O2_ (Torr)^1^.

$$S=\left( {1+23400({P_{O_{2}}}^{3}+150P_{O_{2}})}^{-1} \right)^{-1}$$

1. Calculating a corrected pO2 from measured pO2^2^

$${P_{O_{2}}}_{corrected}={P_{O_{2}}}_{measured}*{10}^{(0.48* \left( pH-7.4 \right)-0.0013*\left( BE \right)+0.024*(37-temp))}$$

1. Calculating Base Excess from pCO2 and pH^3^

$$BE= 0.02786* P_{{CO}_{2}}* {10}^{\left( pH-6.1 \right)}+13.77*pH-124.58$$

Numeric inversions of equations when no trivial algebraic inversion is known were performed using the boost library in the header <boost/math/tools/roots.hpp>. For the example of pH calculation when pCO2 and BE were known, the known value of pCO2 was inserted into formula 3 above, a initial guess of pH = 7.4 was the starting point, and the error function error = f(pH) – BE was defined. Then, the bracket_and_solve_root-function was used to find the pH corresponding to an error of 0.

## Models, libraries and training procedures

The models included are ‘ranger’, ‘xgbTree, ‘svmRadial’, ‘kNN’, ‘RDA’ ‘nnet and ‘glm’ from the ‘caret’-package in R. Other libraries used are ‘data.table’, ‘stringr’, ‘ggplot2’, ‘ggcorrplot’, ‘pROC’, ‘PRROC’, ‘DBI’, ‘stringr’, ‘shapforxgboost’, ’Cairo’, ’ ParBayesianOptimization’.

### Grid search procedure and results

The grids searched and the best hyperparameters found. Grid search was performed using 5-fold repeated cross-validation repeated 5 time with AUROC as metric.

| model | Parameter | set | Best value after grid search |
| --- | --- | --- | --- |
| kNN | k | 3, 5, 7, 9, 11, 13, 15, 17, 19, 21 | 21 |
| RDA | gamma | 0, 0.25, 0.5, 0.75, 1 | 0.5 |
|  | lambda | 0, 0.25, 0.5, 0.75, 1 | 1 |
| xgbTree | nrounds | 400,600 | 600 |
|  | max_depth | 4, 6, 8 | 8 |
|  | eta | 0.1 | 0.1 |
|  | gamma | 0, 0.1 | 0.1 |
|  | colsample_bytree | 0.8, 1 | 0.8 |
|  | min_child_weight | 1,4 | 1 |
|  | subsample | 0.8, 1 | 1 |
| nnet | .decay | 0.75, 0.5, 0.1, 0.01, 0.001, 1e-04, 1e-05, 1e-06 | 0.1 |
|  | .size | 4, 8, 16, 32, 64 | 32 |
| ranger | mtry | 4, 8, 12, 16, 20, 24, 28 | 8 |
|  | min.node.size | 1, 2, 3, 4, 5, 6, 7, 8 | 3 |
|  | splitrule | ‘gini’, ‘extratrees’ | ‘gini’ |
| svmRadial | sigma | 0.00977333188026421, 0.021831312400962, 0.0254247265088816, 0.0444638029482717, 0.0873831748804496 | 0.02542473 |
|  | C | 0.25, 0.5, 1, 2, 4 | 1 |

Supplementary table 4: Grid search space per algorithm

The search grids were based on the default suggestions from the ‘caret’ package, but in certain cases (xgbTree) narrowed due to computational requirements, and in other cases (svmRadial, nnet, ranger) expanded. The sigma values for svmRadial were estimated using the kernlab::sigest-function.

| Algorithm | CV AUROC | Test AUROC | Test AUCPR |
| --- | --- | --- | --- |
| XGBoost | 0,999812 | 0,999884 | 0,998876 |
| Random Forest | 0,999738 | 0,999529 | 0,997094 |
| Neural Network | 0,999634 | 0,999624 | 0,996654 |
| Support Vector Machine | 0,999519 | 0,999491 | 0,996381 |
| Logistic Regression | 0,99859 | 0,999027 | 0,994382 |
| Linear Discriminant Analysis | 0,998351 | 0,998723 | 0,990234 |
| kNN | 0,99623 | 0,994953 | 0,987266 |

Supplementary table 5: Performance of the algorithms in the test set after the grid search procedure

### Forward stepwise feature selection

For the best performing algorithsm (XGBoost and Random Forest), a forward stepwise feature selection process was performed for each algorithms, using the unmodified original data set without the principal components (thus, possibly including correlated features). For algorithms not requiring standardization of variables (xgboost and random forest), no such standardization was performed. For algorithms not requiring a full dataset without missing values (Xgboost), no imputation was performed, otherwise values were imputed with mean or mode, for continuous or categorical features respectively.

The forward feature search was performed using default settings for the algorithms, using 5-fold cross-validation to determine which features to include. This was done until all 39 features were ranked in which order to include in a model for best performance.

### Parameter space during Bayesian optimization phase

After features were ranked using the forward stepwise selection process above, models were sequentially trained on an increasing number of features from 1 up to a maximum of 39. In the Bayesian process, the number of initial random sampling points was 20, and the number of iterations added was 10. 5-fold cross-validated AUROC was the scoring metric in the process. For the best hyperparameters found in the Bayesian process, the full training set was trained and tested with AUCPR on the testing set. If no improvement was seen after 3 consecutive features had been added, further adding of features was considered to be futile, and the best model thus far was considered the best possible model.

In the Bayesian optimization phase, the hyperparameter space searched was defined as follows:

| algorithm | Parameter | Type | Range |
| --- | --- | --- | --- |
| xgbTree | max_depth | Integer | 3 – 10 |
|  | eta | Real | 0.01 – 0.2 |
|  | gamma | Real | 0-10 |
|  | colsample_bytree | Real | 0.6-1 |
|  | min_child_weight | Integer | 1-40 |
|  | max_delta_step | Integer | 1-40 |
|  | lambda | Real | 0-1 |
| ranger | splitrule | Factor | ‘gini’, ‘extratrees’ |
|  | min_node_size | Integer | 1-50 |
|  | mtry | Integer | 1-11 |
|  | ntrees | Integer | 100-5000 |

Supplementary table 6: Hyperparameter range during Bayesian optimization

At any given step, the parameter ‘mtry’ for ranger was kept at less or equal to the number of features in the model. The parameter ‘nrounds’ for xgboost was determined through cross-validation, with ‘nrounds’ initially at 1000 and ‘early_stopping_rounds’ at 50, and the final ‘nrounds’ determined by ‘best_iteration’ from the xgb.cv-function.

## Results from Bayesian optimization

### Random forest

| algorithm | nfeats | test_auc | test_aucpr |
| --- | --- | --- | --- |
| ranger | 1 | 0.988506545098311 | 0.971933993562633 |
| ranger | 2 | 0.991714598916136 | 0.981517164045733 |
| ranger | 3 | 0.998461539780125 | 0.994246367893065 |
| ranger | 4 | 0.99974523270175 | 0.997664502580413 |
| ranger | 5 | 0.99986822381125 | 0.998684313893426 |
| ranger | 6 | 0.998729163291911 | 0.996590034743309 |
| ranger | 7 | 0.999720163085354 | 0.997748351211293 |
| ranger | 8 | 0.999879151592756 | 0.998853999913225 |
| ranger | 9 | 0.99767409670101 | 0.993654417439581 |
| ranger | 10 | 0.999811013661013 | 0.998357920928969 |
| ranger | 11 | 0.999721020166256 | 0.997601212461345 |
| ranger | 12 | 0.999761731509122 | 0.997979342942036 |

Supplementary table 7: Results of forward stepwise feature selection and Bayesian optimization, for Random Forest

The final parameters for the ranger model with 8 predictors was splitrule = ‘extratrees’, min.node.size = 3, mtry = 4 and num.trees = 1394.

### Xgboost

| algorithm | nfeats | test_auc | test_aucpr |
| --- | --- | --- | --- |
| xgbTree | 1 | 0.992377550994171 | 0.983255729826641 |
| xgbTree | 2 | 0.999157918013355 | 0.992734287888497 |
| xgbTree | 3 | 0.999669166771659 | 0.996969750373476 |
| xgbTree | 4 | 0.999737518973629 | 0.99751996764677 |
| xgbTree | 5 | 0.999750803727616 | 0.99746659431445 |
| xgbTree | 6 | 0.999847011058915 | 0.998515055537439 |
| xgbTree | 7 | 0.999855153327488 | 0.998581725628365 |
| xgbTree | 8 | 0.99985601040839 | 0.998633038450662 |
| xgbTree | 9 | 0.999905721100732 | 0.999074711645224 |
| xgbTree | 10 | 0.999875723269147 | 0.998825623883132 |
| xgbTree | 11 | 0.999867581000573 | 0.99873767236425 |
| xgbTree | 12 | 0.999855153327488 | 0.998637099335908 |
| xgbTree | 13 | 0.999899292993964 | 0.999008663396068 |

Supplementary table 8: Results of forward stepwise feature selection and Bayesian optimization, for XGBoost

The final parameters for the xgboost model with 9 features were booster = ‘gbtree’, max_depth = 9, eta = 0.1381765, gamma = 0, colsample_bytree = 0.8, min_child_weight = 1, max_delta_step = 0, lambda = 1.

## Summary of missclassifications from XGBoost model

In our study, there were four misclassified cases among the samples originally marked as arterial. All had been manually assessed by both domain experts (prior to any model development) to establish the reference label. Importantly, all errors occurred during clinically complex scenarios and required thorough manual review of the information in the EHR (beyond the blood gas parameters themselves) to establish the true sample type.

Cases 1 and 2: Both samples were taken from the same patient during ongoing cardiopulmonary resuscitation during hypoxic cardiac arrest. The PaO2-values were around 1.0 kPa, rarely seen in true arterial samples in real-life settings, and far lower than any values present in the training data. These outlier values in physiological parameters likely contributed to the misclassification.

Case 3: This sample came from a patient experiencing rapid respiratory and circulatory deterioration. The PaO2 was 4.1 and SaO2 was 45%, while the peripheral saturation was 64%, all substantially lower than preceding samples. The patient transitioned from spontaneous breathing to mechanical ventilation with FiO2 of 100% in less than two hours, had profound and refractory shock, and the blood gas sample was taken during this session of deterioration. The abrupt shift in physiology may have influenced the model to misclassify this arterial sample as venous.

Case 4: This patient was in severe hypoxic respiratory failure and shock, receiving 100% FiO2, had hypothermia, and ongoing transfusion requirement due to bleeding and rapidly rising plasma lactate. The marked changes in acid-base status and oxygen transport capacity likely altered oxygen dissociation dynamics, contributing to the misclassification.

These 4 cases all required thorough manual review of EHR data to determine true sample type and highlight the model’s limitations during extreme fluctuations in physiological parameters in patients with extremely severe respiratory and circulatory derangement - especially during scenarios where the peripheral saturation may be unmeasurable due to impending or actual cardiac arrest.

## Confusion matrices

| Random Forest (Bayesian Optimization) | full | predicted class | | marked arterial | predicted class | |
| --- | --- | --- | --- | --- | --- | --- |
|  | True class | v | a | True class | v | a |
|  | v | 597 | 31 | v | 13 | 0 |
|  | a | 5 | 6181 | a | 5 | 6177 |
| XGBoost (Bayesian Optimization) | full | predicted class | | marked arterial | predicted class | |
|  | True class | v | a | True class | v | a |
|  | v | 602 | 26 | v | 13 | 0 |
|  | a | 8 | 6178 | a | 4 | 6178 |
| Logistic Regression | full | predicted class | | marked arterial | predicted class | |
|  | True class | v | a | True class | v | a |
|  | v | 590 | 38 | v | 13 | 0 |
|  | a | 20 | 6166 | a | 12 | 6170 |
| Random Forest | full | predicted class | | marked arterial | predicted class | |
|  | True class | v | a | True class | v | a |
|  | v | 597 | 31 | v | 13 | 0 |
|  | a | 10 | 6176 | a | 1 | 6181 |
| Neural Network | full | predicted class | | marked arterial | predicted class | |
|  | True class | v | a | True class | v | a |
|  | v | 594 | 34 | v | 13 | 0 |
|  | a | 11 | 6175 | a | 2 | 6180 |
| Regularized Linear Discriminal Analys | full | predicted class | | marked arterial | predicted class | |
|  | True class | v | a | True class | v | a |
|  | v | 520 | 108 | v | 13 | 0 |
|  | a | 7 | 6179 | a | 14 | 6168 |
| kNN | full | predicted class | | marked arterial | predicted class | |
|  | True class | v | a | True class | v | a |
|  | v | 544 | 84 | v | 12 | 1 |
|  | a | 9 | 6177 | a | 4 | 6178 |
| Support Vector Machine | full | predicted class | | marked arterial | predicted class | |
|  | True class | v | a | True class | v | a |
|  | v | 597 | 31 | v | 13 | 0 |
|  | a | 9 | 6177 | a | 1 | 6181 |
| XGBoost | full | predicted class | | marked arterial | predicted class | |
|  | True class | v | a | True class | v | a |
|  | v | 599 | 29 | v | 13 | 0 |
|  | a | 6 | 6180 | a | 1 | 6181 |
| XGBoost (DART) | full | predicted class | | marked arterial | predicted class | |
|  | True class | v | a | True class | v | a |
|  | v | 598 | 30 | v | 13 | 0 |
|  | a | 9 | 6177 | a | 2 | 6180 |
| SO2 - SpO2 matched difference | full | predicted class | | marked arterial | predicted class | |
|  | True class | v | a | True class | v | a |
|  | v | 546 | 82 | v | 11 | 2 |
|  | a | 6 | 6180 | a | 3 | 6179 |

Supplementary Table 9: Confusion matrices for all algorithms in the holdout set

## Manual review

Manual review of blood gas classifications was conducted by a specialist physician in anesthesia and intensive care, with extensive experience in interpreting blood gas results in critically ill patients. The reviewer assessed each patient’s blood gases in a spread sheet sorted by sample time, to aid in assessment of trends of parameters. The review utilized routinely available parameters including pH, PO_2_, PCO_2_, base excess (BE), HCO_3_^-^, lactate, O_2_ saturation, hematocrit and hemoglobin, in conjunction with contextual information such as peripheral SpO_2_, FiO_2_, and whether the sample was initially labeled as arterial or venous. In many cases, the information contained in the absolute values and trends of these parameters was sufficient to determine true sample type with certainty.

In ambiguous cases, where further information was needed to be certain of sample type, contextual clues such as sample frequency, trends of ventilator settings such as FiO2, trends in MAP values, recorded procedures and diagnoses and other clinical data was used at the discretion of the reviewer. This clinical classification process reflects a level of contextual integration that is challenging to capture in rule-based systems, underscoring the value of expert manual annotation for algorithm development and validation.

A second specialist physician in anesthesia and intensive care reviewed all ambiguous cases, all marked erroneously and all arterial with a registered PO_2_ < 6.66 kPa (50 mmHg). Consensus was reached in labeling all blood gas sample types.

## References

1. Severinghaus JW. Simple, accurate equations for human blood O2 dissociation computations. *J Appl Physiol Respir Environ Exerc Physiol*. Mar 1979;46(3):599-602. doi:10.1152/jappl.1979.46.3.599

2. Kelman GR, Nunn JF. Nomograms for correction of blood Po2, Pco2, pH, and base excess for time and temperature. *J Appl Physiol*. Sep 1966;21(5):1484-90. doi:10.1152/jappl.1966.21.5.1484

3. Siggaard-Andersen O. An acid-base chart for arterial blood with normal and pathophysiological reference areas. *Scand J Clin Lab Invest*. May 1971;27(3):239-45. doi:10.3109/00365517109080214
